# Modulation of Nrf2/HO-1 by Natural Compounds in Lung Cancer

**DOI:** 10.3390/antiox12030735

**Published:** 2023-03-16

**Authors:** Somayyeh Ghareghomi, Faezeh Moosavi-Movahedi, Luciano Saso, Mehran Habibi-Rezaei, Ali Khatibi, Jun Hong, Ali A. Moosavi-Movahedi

**Affiliations:** 1 Institute of Biochemistry and Biophysics, University of Tehran, Tehran 1417466191, Iran; ghareghomi.s@ut.ac.ir (S.G.); fmoosavi@ut.ac.ir (F.M.-M.); 2 Department of Physiology and Pharmacology “Vittorio Erspamer”, Sapienza University of Rome, 00185 Rome, Italy; 3 School of Biology, College of Science, University of Tehran, Tehran 1417466191, Iran; 4 Center of Excellence in NanoBiomedicine, University of Tehran, Tehran 1417466191, Iran; 5 Department of Biotechnology, Faculty of Biological Sciences, Alzahra University, Tehran 1993893973, Iran; khatibi@alzahra.ac.ir; 6 School of Life Sciences, Henan University, Kaifeng 475000, China; hongjun@henu.edu.cn; 7 UNESCO Chair on Interdisciplinary Research in Diabetes, University of Tehran, Tehran 1417466191, Iran

**Keywords:** oxidative stress, Keap/Nrf2 pathway, Heme oxygenase-1, antioxidants, lung cancer

## Abstract

Oxidative stresses (OSs) are considered a pivotal factor in creating various pathophysiological conditions. Cells have been able to move forward by modulating numerous signaling pathways to moderate the defects of these stresses during their evolution. The company of Kelch-like ECH-associated protein 1 (Keap1) as a molecular sensing element of the oxidative and electrophilic stress and nuclear factor erythroid 2 (NF-E2)-related factor 2 (Nrf2) as a master transcriptional regulator of the antioxidant response makes a master cytoprotective antioxidant pathway known as the Keap1/Nrf2 pathway. This pathway is considered a dual-edged sword with beneficial features for both normal and cancer cells by regulating the gene expression of the array of endogenous antioxidant enzymes. Heme oxygenase-1 (HO-1), a critical enzyme in toxic heme removal, is one of the clear state indicators for the duality of this pathway. Therefore, Nrf2/HO-1 axis targeting is known as a novel strategy for cancer treatment. In this review, the molecular mechanism of action of natural antioxidants on lung cancer cells has been investigated by relying on the Nrf2/HO-1 axis.

## 1. Introduction

Cellular metabolism has always been associated with the production of functional intermediates and reactive oxygen/nitrogen species (ROS/RNS). These species must be removed from the intracellular environment immediately due to their highly destructive capacity on biomolecules. Destructive changes in DNA, proteins, and lipids have usually been involved in a variety of diseases, including cancer, inflammation, and neurodegenerative diseases [1,2,3]. The hemostatic condition of the cells guarantees the continuation of normal cell function, including the activation of a variety of antioxidant pathways to adjust the cellular oxidative conditions. The companionship of Kelch-like ECH-associated protein 1 (Keap1) with highly reactive cysteine residues serves as a molecular sensing element of oxidative and electrophilic stresses, and nuclear factor erythroid 2 (NF-E2)-related factor 2 (Nrf2), a bZip transcription factor, as a master transcriptional regulator of the antioxidant response forms a master cytoprotective antioxidant pathway known as the Keap1/Nrf2 pathway [4]. In normal conditions, the Keap1/Nrf2 complex Keap1, as an adaptor subunit of cullin 3-based E3 ubiquitin ligase, directs Nrf2 for proteasomal degradation [5]. In contrast, under oxidative stress conditions, the reactive cysteine residue modification leads to the release of Nrf2 to translocate to the nucleus. In the nucleus, Nrf2 binding to antioxidant response elements (ARE) induces the expression of a wide variety of cytoprotective enzymes such as superoxide dismutases (SOD), catalase (CAT), NAD(P)H quinone dehydrogenase 1 (NQO1), and glutathione S-transferases (GSTs) [6].

**Figure 1:**
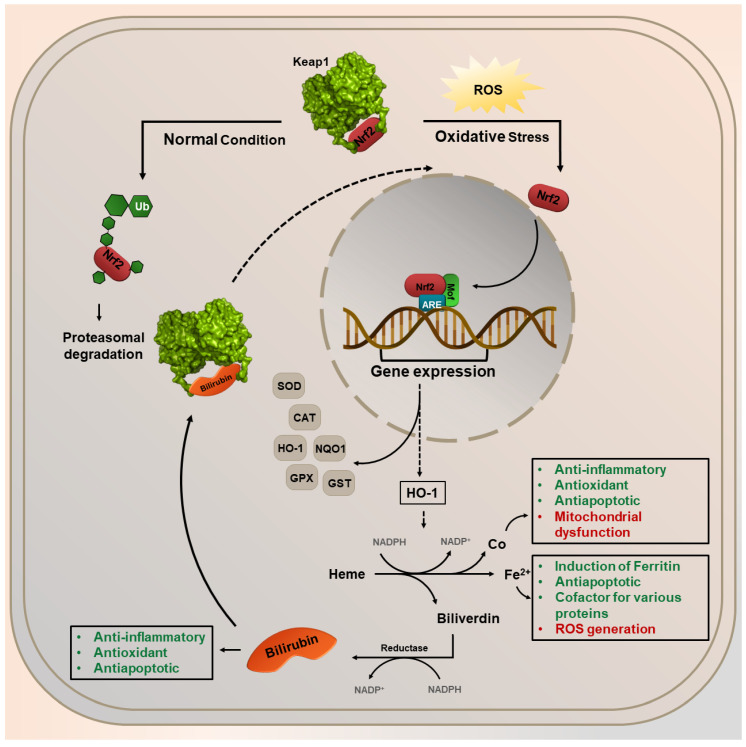
Nrf2/HO-1 axis activity in oxidative conditions. Nuclear localization of Nrf2 in oxidative conditions triggers several antioxidative proteins. HO-1 is an essential enzyme in the degradation of heme and the production of some functional components. These compounds display various beneficial (in green) and harmful (in red) effects on cells.

Nrf2 activation is a dual-edged sword; the activation of Nrf2 is a crucial event in normal cell homeostasis, but in cancer cells supports their rapid growth, proliferation, and drug resistance [7]. Heme oxygenase-1 (HO-1) is one of the three isoforms of HO, the most basic enzyme in the degradation of heme to free iron, carbon monoxide (CO), and biliverdin [8] (Figure 1). Based on various studies, HO-1 has been introduced as a pivotal player in Nrf2-mediated cancer cell survival and resistance against anticancer therapies [9,10]. Therefore, detailed molecular investigations on the function of HO-1 in normal and cancer cells, mainly through the HO-1 action products, are of particular importance. Accurate determination of the effects of these products and their modulation by various agents is considered one of the novel and practical strategies for targeting cancer cells. In this study, we focused on Nrf2/HO-1 axis modulation by natural compounds in lung cancer treatment.

## 2. ROS and Its Pivotal Role in Cells

**Figure 2:**
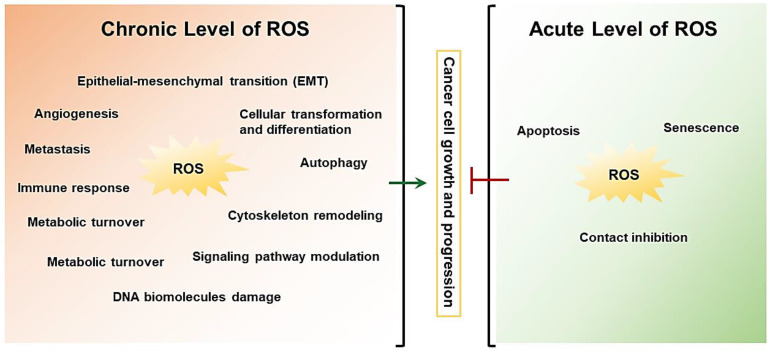
Two different edges of ROS in various biological pathways. ROS at chronic and acute levels can promote several pathways. In normal cells, a high level of ROS can create oxidative conditions and damage cells. This event is the basis for the occurrence of various mutations and the creation of cancerous cells. In cancer cells, acute levels of ROS can create unfavorable conditions and conduct cancer cells to apoptosis and death.

Reactive oxygen species (ROS), including O_2_^•−^, HO^•^, and H_2_O_2_, are generally specified as oxygen-containing chemical species with reactive chemical properties. They are continuously created through both enzymatic and non-enzymatic reactions in a biological system [11]. At first glance, these molecules as destructive agents must be eliminated from the cells, which is done through antioxidant pathways [12]. However, in the evolutionary pathway or adaptation of cells to the existing conditions, ROS display a new view of themselves in biological activities as signaling molecules, which is very important; due to their unique properties, they can initiate various pivotal pathways in cells that ultimately control a variety of cell functions to control development, differentiation, redox levels, stress signaling, interactions with other organisms, cell death, and drug resistance. The obvious role of ROS in various disease development and its involvement in several mechanisms have been confirmed through numerous studies. Due to the high metabolic activity of cancer cells, excessive production of ROS is inevitable. ROS, by affecting various cellular processes, provide desired conditions for the growth and progression of cancer cells. As shown in Figure 2, in oxidative conditions, the antioxidant responses of cell hemostasis are established, but in acute oxidative conditions, the induction of cell death through multiple mechanisms will lead to the removal of cancer cells [13,14]. This condition is exactly what many chemotherapy drugs have created and, in this way, prevents the growth and proliferation of cancer cells. The mentioned process has been investigated in various studies, and the obtained results confirm the role of ROS created in cells in inducing cell death.

Otherwise, oncogenic signals may trigger the generation of ROS in cancer cells [15]. The role of some oncogenes, such as *c-Myc* and k-*Ras*, in increasing the production of ROS in cells has been proven [16,17]. The accumulation of ROS in the cancer cell can increase the destructive effect on DNA, proteins, and other biomolecules. Therefore, an increase in oxidized products has been identified in a variety of cancers [18,19,20,21]. In the first step, cells increase their antioxidant capacity through increased gene expression and protein levels of various enzymatic and non-enzymatic proteins involved in antioxidant responses and maintain cellular redox balance [22,23,24]. However, in some cancers, its expression and activity were reported to be constant [25]. Therefore, heterogeneity was observed in various cancers in terms of the level of ROS and how to respond to these oxidative stresses.

Various mechanisms are observed for the production of ROS in cancer cells; besides the cellular electron transfer chain (ETC) in the mitochondria as the most important production site of ROS generation, the activity of different oncogenic pathways has also been reported. The oncogene *c-Myc* can induce DNA damage and decrease p53 activity through ROS production [16]. The oncogenic *Ras2* (val19) allele, through activation of the cAMP-PKA pathway, increases the production of ROS and enhances oxidative protein damage [26]. In addition, there is an association between *Ras* oncogene and the production of superoxide anion extracellularly through a membrane-associated NADPH oxidase. The generation of superoxide radicals in *Ras*-transformed cells is essential for their proliferation and the maintenance of the transformed state [27,28].

Meanwhile, a significant pathway in cancer cells is the increased ROS level through an established defective ETC upon mutation of exon sequences for coding the proteins on mitochondrial DNA (mtDNA) for the respiratory chain components to bring about the enhanced production of ROS and its consequences [29,30,31,32,33]. ROS stress induces a wide range of biological responses, from growth and proliferation to senescence and cell death [34]. On the other hand, due to the very high amount of ROS produced, the antioxidant capacity of the cell is minimized, which leads to the sensitivity of these cells to chemotherapy agents. Besides this, ROS is a fundamental messenger in the promotion of various signaling pathways involved in cell growth and proliferation [28]. In this aspect, ROS can stimulate cell growth and proliferation through direct interaction with certain receptors and modulation of the redox conditions of signaling molecules, such as several protein kinases and transcription factors. Oxidation of thioredoxin (TRX) causes its dissociation from apoptosis signaling kinase-1 (ASK1), which is then activated by *tumor necrosis factor* (TNF) receptor-associated factor 2 (TRAF2), causing the activation of SAPK/JNK [35]. Therefore, ROS, through modulating the mitogen-activated protein kinase (MAPK) cascade, a key regulatory pathway in many facets of cellular functions, including metabolic regulation and cell proliferation, can trigger cell proliferation and development [36]. Oxidative modification of various transcription factors and protein kinases such as nuclear factor kappa-light-chain-enhancer of activated B cells (NF-κB), hypoxia-inducible factor (HIF-1α), protein kinase C (PKC), and extracellular signal-regulated kinase (ERK) has been revealed to be contained in ROS-mediated modulation of cell survival and progression [37,38]. On the other hand, oxidative modulation of p53, as a tumor suppressor, can disrupt the cell-cycle regulatory function of p53 and donate to unrestrained cell growth and propagation [39]. Based on numerous studies, ROS able to attack various components of DNA and create some modified products such as oxidized bases, strand breaks, intra-strand adducts, and DNA–protein crosslinks [40]. Therefore, the production of ROS in mitochondria is considered the main source of intracellular destructive agents, and as mentioned, the sensitivity of mtDNA is much higher than that of nuclear DNA [41]. In addition to DNA, proteins and membrane lipids are also affected by ROS, which results in disrupting the normal function of cells [42]. The activity of some specific protective proteins, such as CAT and peroxidase (POX), is reduced by their oxidation, and this leads to a decrease in the antioxidant capacity of cells in dealing with oxidative stress.

In parallel with oxidation, nitrosylation of protein is another mechanism of cellular damage. Various signaling molecules such as NF-κB, activator protein 1 (AP-1), and tumor suppressor p53 are affected by ROS through nitrosylation, which in turn will lead to a change in their function [43,44].

ROS-mediated lipid peroxidation reduces the fluidity of the biological membranes and increases the membrane penetrability. In the mitochondria, this will lead to the release of cytochrome c (Cyt c) from the mitochondrial intermembrane space (IMS) and initiate the apoptosis pathway upon binding to apoptotic protease activating factor 1 (Apaf-1) to organize a heptameric complex known as the apoptosome, as the main mechanism of inducing apoptosis by ROS [13,45]. However, massive cellular oxidation by high levels of ROS can trigger necrosis instead of apoptosis, or a combination of apoptosis and necrosis occurs in cells after extensive destruction [46].

According to past studies, the increase of ROS in cancer cells above the threshold leads to more sensitivity of cells to chemotherapy drugs and increases the efficiency of treatment [47,48]. However, it has been recently accepted that cells in chronic oxidative conditions can become drug resistant by increasing the activity of special antioxidant pathways, which reduces their sensitivity against chemotherapy [49,50]. Therefore, ROS plays different roles in chronic and acute oxidative conditions indicating these species lead different functions in the cell by stimulating different molecular mechanisms according to cell conditions [7]. Considering the intense basal metabolism in cancer cells and their need for excessive energy, they are often in a chronic oxidative condition, which leads to the initiation of a series of processes that will benefit the cancer cell altogether.

## 3. Master Antioxidant Pathway Induced in Oxidative Stress Condition

Cells need an antioxidant defense barrier against oxidizing molecules to establish cell hemostasis. Antioxidants are nucleophiles that have a high affinity to react with electrophilic reactive species and neutralize them. Glutathione (GSH), as the most abundant endogenous antioxidant molecule, displays a specific role in the oxidative response. It can effectively eliminate ROS and decreases the generation of the oxidative signal. In this process, GSH donates an electron to form two oxidized GSH (GSSG) [51,52]. GSH, through revitalizing other antioxidant enzymes, displays a specific indirect role in antioxidant defense [53]. Sensing ROS and adjusting multiple pathways to respond to them is the first step in dealing with oxidant species. So far, about 20 transcription factors have been identified that are sensitive to redox conditions. These transcriptional factors are essential for creating a balanced oxidative condition in cells. Forkhead box class O (FOXO) and Nrf2 are two master opposing pathways against cellular oxidative stress.

### 3.1. FOXO Pathways Activation and Its Consequences

FOXO transcription factors are parts of the Forkhead box family of transcription factors, including FOXO1, FOXO3a, FOXO4, and FOXO6 [54]. They are involved in various cellular functions, including cell proliferation, apoptosis, differentiation, modulation of oxidative stress, and DNA damage [55,56].

**Figure 3:**
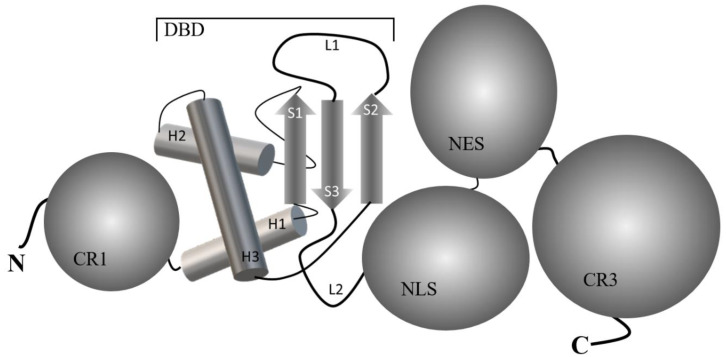
Common five-domain structure/organization of FOXO proteins. The graphical structure of the DNA binding domain (DBD) from FOXO1 has been presented based on the solution NMR method (PDB-ID 6QVW). H1, H2, and H3 are helices, S1, S2, and S3 are strands, and L1 and L2 are wing-like loops. CR1 and CR2 depict N-terminal and C-terminal region domains, respectively. Nuclear localization signal (NLS) and nuclear export sequence (NES) domains are also depicted.

Figure 3 shows a common five-domain structure/organization of FOXO proteins, and their arrangement in the sequence has been elucidated: N-terminal region (CR1), winged-helix DNA-binding domain (DBD) known as Forkhead domain ((PDB ID 6QVW), nuclear localization sequence (NLS), nuclear export signal (NES) and C-terminal conserved regions (CR3), also known as transactivation domain (TAD) [57]. DBD consists of about 110 amino acid residues, which are folded at the secondary structure level into three α-helices (H1, H2, and H3), a three-stranded antiparallel β-sheet comprising three twisted strands (S1-S2 and S3), and two wing-like loops (L1 and L2) which are arranged H1-S1-H2-H3-S2-L1-S3-L2 sequence [56]. Among them, the helix H3, in collaboration with loops, is responsible for recognizing and specifically binding to “forkhead-responsive DNA elements” (FHRE) with a core consensus sequence 5′-(A/C)AA(C/T)A-3′ in the major groove of the DNA [58].

**Figure 4:**
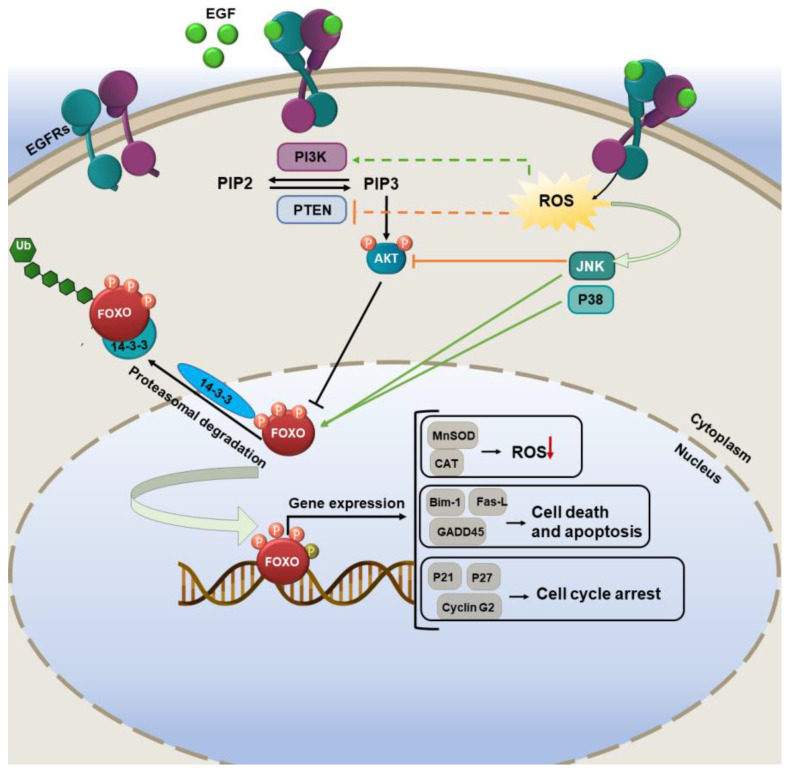
The FOXO pathway regulation by ROS. In oxidative conditions, phosphorylation of FOXO family members by various upstream effectors can trigger some antioxidant enzyme expression and reduce ROS levels in cells.

Several pieces of evidence show that the deregulation of FOXO proteins is connected with tumorigenesis and cancer development. Based on various studies, FOXO factors are both sensors of oxidative stress signals and effectors of the consequent cellular response. FOXO proteins control the intracellular redox environment through some mechanisms. Activation of FOXO increases manganese-dependent SOD (MnSOD or SOD2) and CAT, as well as other opposing proteins against oxidative stress, such as sestrin 3 and PTEN-induced kinase 1 (PINK1) [59]. ROS activates FOXO indirectly or directly. Indirect activation operates via the phosphorylation of FOXO by activated c-Jun N-terminal kinases (JNK) and p38 MAPK cascades [60] (Figure 4). Direct FOXO modification through targeted sulfhydryl group (−SH) oxidation on FOXO to generate disulfides or persulfides [61].

Thioredoxin binding protein (TXNIP) has multiple functions and plays an important role in redox homeostasis [62]. FOXO, through modulating TXNIP, can promptly activate thioredoxin toward reducing the cellular ROS levels in glucose-treated endothelial cells [63]. The interaction of FOXO1 with sirtuin 1 (SIRT1) under oxidative conditions led to the triggering of anti-stress-related genes, thus boosting the growth and survival of the cells [64]. Based on some studies, FOXO has a potent correlation with *p53* in cell cycle regulation and tumor suppression [65,66]. *p53* can directly target FOXO3a and increases its nuclear translocation and induction of apoptosis. FOXO can trigger the induction of autophagy in cells through the upregulation of various autophagy-related genes such as autophagy-related genes (Atg4, Atg7, and Atg14) [67,68].

### 3.2. Nrf2 Pathway Activation and Its Consequences

**Figure 5:**
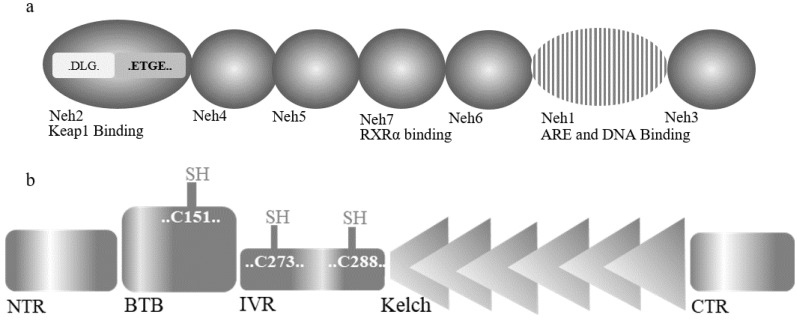
Domain structures of (**a**) Nrf2 and (**b**) Keap1.

Nrf2, as a pivotal redox-sensitive transcription factor, controls the expression of an array of antioxidant proteins and plays a fundamental role in antioxidant response. Nrf2 contains 605 amino acids that are classified into seven efficient *Nrf2*-ECH homology domains (Neh1-7) [12,69] (Figure 5a). Neh1 is involved in ARE and DNA binding; moreover, Neh1 and Neh6 have the main role in regulating Nrf2 stability [70]. The Neh2 domain, at the N-terminal, comprises seven lysine residues responsible for ubiquitination, and ETGE and DLG motifs involve in Keap1 binding [71]. Interaction of Neh3, Neh4, and Neh5 with coactivators can prompt the activation of Nrf2-related genes. Finally, Neh7, through binding to retinoic X receptor α (RXRα), an Nrf2 inhibitor, can negatively regulate Nrf2-related genes [72].

Kelch-like ECH-associated protein 1 (Keap1), a zinc-metalloprotein, is the essential regulator of Nrf2 activity [73]. Keap1 protein is composed of 624 amino acids and has five structural regions: N-terminal region (NTR), common complex/tramtrack/bric-a-brac (BTB) domain, intervening region (IVR), Kelch domain, and C-terminal region (CTR) (Figure 5b). BTB domain mediates the homodimerization of Keap1 and Cullin3 (Cul3). Keap1 also has 27 cysteine residues, among them with more than 20 free sulfhydryl groups [74,75]. These highly reactive groups act as stress sensors in the oxidative condition where modification of these residues can modulate its function. Cys273 and Cys288 are the most important residues for Keap1 to regulate Nrf2 under normal and stress conditions, while Cys151 in the BTB domain is generally recognized as the most necessary sensory element under oxidative conditions [76,77,78].

In normal conditions, Keap1 binds to Nrf2 in the cytosol and, through forming a complex with cullin3 (CUL3), catalyzes the poly-ubiquitination of Nrf2 and its degradation [79]. Under oxidative stress conditions, the disassociation of Nrf2 and Keap1 and the nuclear translocation of Nrf2 leads to the upregulation of various cytoprotective genes [80]. Furthermore, modulation of Nrf2 activity can occur through the phosphorylation of several Ser/Thr residues by numerous kinases, including MAPKs, PI3K/AKT, and PKC [81]. Nrf2-related oxidative response in cells involves boosting the expression of various antioxidant enzymes such as SOD, CAT, HO-1, thioredoxin reductase (TrxR), glutathione reductase (GR), and NAD[P]H quinone dehydrogenase-1 (NQO-1) [12].

The SODs are a family of enzymes that efficiently catalyze the dismutation of the superoxide radical anion and produce hydrogen peroxide (H_2_O_2_) and H_2_O. Three isoforms of this enzyme comprise the cytosolic-CuZnSOD (*Sod1*), mitochondrial superoxide dismutase MnSOD (*Sod2*), and the extracellular secreted enzyme, which is attached to proteoglycans and the cell surface [82]. Exposure to H_2_O_2_ is a generally used process to trigger oxidative damage in cells. The Fenton reaction between H_2_O_2_ and Fe^2+^ ions increases hydroxyl radical levels and is a central mechanism for the exacerbation of oxidative damage. The peroxide formed is modified by the enzymes of the glutathione redox cycle and catalase [83].

Several studies have depicted that the activation of the thioredoxin (Trx) system comprising Trx, NADPH, and thioredoxin reductase (TrxR) in particularly TrxR1, is important for counteracting Nrf2 activation [84]. These observations are strongly suggestive of direct functional links between TrxR1 and Nrf2 [85]. In mammalian cells, there are two main thiol-dependent antioxidant systems, a 12 kDa oxidoreductase protein, thioredoxin (Trx), and the most abundant non-protein thiol-harboring molecule, glutathione (GSH). The Trx active site contains two key cysteine residues in a CXXC motif able to reversibly oxidize into a dithiol and reduce other substrate proteins as an essential component of the redox activity of the Trx system. This system, through providing electrons for various enzymes, exerts a fundamental function in the protection of DNA against oxidative stress [86].

According to previous studies, hyperactivation of Nrf2 can initiate multiple signaling pathways in the cells and is involved in proliferation, survival, metabolic reprogramming, angiogenesis, drug resistance, and metastasis [7]. Therefore, a detailed examination of the antioxidant elements in the Nrf2 pathway is very important to understand their function in cells under acute or chronic stress conditions.

HO-1 has been identified as a functional effector of Nrf2-related cell responses [8,87]. Some evidence identified that this stress protein plays a different role in normal and cancer cells. This means that some of the oncogenic activities of Nrf2 in cancer cells may be the results of the activity of this enzyme [9]. Therefore, in this review, we have focused on the Nrf2/HO-1 axis overlapping mechanisms and its targeting by natural compounds to modulate cancer cell growth and survival.

## 4. Nrf2 and HO-1 Expression Overlapping for the Benefit or Loss of Cells

Nrf2 is a transcription factor with plenty of known target genes, including heme oxygenase-1 (HO-1, EC 1.14.99.3), whose expression is under its control. HO-1 is one of two HO isoforms found in mammals. It presents as an inducible 32 kDa protein and is extremely upregulated by numerous stimuli like heme, nitric oxide, heavy metals, growth factor, and modified lipids [88]. This cytoprotective enzyme catalyzes the rate-limiting step in heme degradation, leading to the production of equimolar extents of iron ions, biliverdin, and carbon monoxide [89]. These products play an important role in biological processes, including fibrosis, inflammation, apoptosis, angiogenesis, cell growth, and cell proliferation. Additionally, the activity of HO-1 and its cytoprotective by-products can create a remarkable benefit for tumor cells to overwhelm the enhanced oxidative stress during tumorigenesis. On the other hand, the overexpression of HO-1 has also been reported to benefit cancer cells and trigger their growth, survival, and migration [90]. Therefore, depending on the physiological conditions, the function of HO-1 and its products can exert different effects on cells.

Just like normal cells, in tumor cells, HO-1 products regulate inflammatory responses and mediate immunosuppression [91,92]. The expression of HO-1 in lung cancer tumors is about ∼4.7-fold as compared with normal tissue. Based on some studies, HO-1, due to its cytoprotective activity, can interrupt tumor initiation; however, similar properties may stimulate tumor proliferation [93]. On the other hand, stable overexpression of HO-1 in murine C2C12 myoblasts leads to the resistance of these cells to ROS and their high proliferative power [94].

Studies have shown that the cytoprotective activity of HO-1 is in close correlation with heme metabolism, including biliverdin (BV) and bilirubin (BR), with a high capacity of the antioxidant system for scavenging ROS. The BV/BR system also suppresses lipid and protein peroxidation, and even in this respect, it can compete with antioxidant vitamin E [95,96]. BV, through modulation of various enzymes, can exert a modulatory effect on some signaling pathways, such as AKT and MAPK signaling and the activation of endothelial NO synthase, and display its cytoprotective properties in normal and cancer cells [97,98]. BV also has an essential role in inducing apoptosis in a concentration-dependent manner; a very high level may induce apoptosis; on the contrary, a low level of BV inhibits apoptotic pathways [99,100]. Moreover, BV is a specific inducer of proangiogenic vascular endothelial growth factor (VEGF) and interleukin 8 (IL-8) expression in human keratinocytes, and this function depends on the cell type in a concentration-dependent manner [89,101]. Based on various in vivo and in vitro studies, there is a reverse relationship between serum BV levels and various cancer incidences such as lung, breast, and colorectal cancers [102,103].

Ferrous is one of the main products of HO-1 activity that can exert a toxic effect on cells through interaction with cellular oxidants to generate oxidative conditions [104]. In addition, various molecular mechanisms of the tumorigenic result of iron in tumor cells might be reliant on its genotoxic properties and the modulation of the Wnt signaling pathway [105]. The induction of HO-1 expression in endothelial cells exposed to H_2_O_2_ is attended by the induction of ferritin, a protective molecule sequestering iron ions-induced oxidative stress [106]. The Fe-ATPase pump regulates the free iron content in cells [107]. Angiogenesis, the formation of new blood vessels, is one of the pivotal hallmarks of cancer cells and was described in 1971 by Judah Folkman [108]. It makes it possible to supply nutrients and oxygen for cancer cells and eliminates metabolic wastes and CO_2_. According to some studies, the induction of ferritin exerts a specific role in angiogenesis. Ferritin has a main role in angiogenesis which was first proposed by Coffman et al. in 2009. Based on this study, ferritin binds to cleaved high molecular weight kininogen (Hka) with high affinity (K_d_~13 nM) and antagonizes the antiangiogenic effects of HKa, triggering the migration, assembly, and survival of HKa-treated endothelial cells [109]. Therefore, using various types of iron chelators as therapeutic agents can be one of the ways to deal with its cytotoxic effect in cancer cells.

Carbon monoxide (CO), as another product, was generally revealed to facilitate numerous useful HO-1 effects, counting as a defense agent against oxidative stress, apoptosis, inflammation as well as angiogenesis. CO shows a proangiogenic effect in cancer cells and various angiogenic factors, like VEGF and stromal cell-derived factor-1 (SDF-1), and facilitates its pro-angiogenic properties by the stimulation of HO-1 activity [110]. CO also displays a main role in the modulation of cancer cell metabolism by enhancing VEGF synthesis in various cell types [111]. On the other hand, the response of endothelial cells to the stimulation of VEGF and SDF-1 needs the expression of HO-1 [112]. Based on some studies, stimulation of VEGF expression by CO is related to the activation of hypoxia-inducible factor-1 (HIF-1α) [113]. In contrast, in some cancers, such as non-small-cell lung carcinoma (NSCLC) and prostate cancers, overexpression of HO-1 is associated with inhibiting angiogenesis mediators [114,115]. On the other hand, HO-1/CO displays an important regulatory effect on cancer cell metabolism. CO-sensitive methylation of PFKFB3 (6-Phosphofructo-2-Kinase/Fructose-2,6-Biphosphatase 3), an enzyme producing fructose 2,6-bisphosphate (F-2,6-BP), acts as an activator of phosphofructokinase-1, which is a rate-limiting glycolytic enzyme. HO-1 stimulation or CO production results in decreased methylation of PFKFB3 in various cancer cells to repress F-2,6-BP, altering glucose consumption from glycolysis toward the pentose phosphate pathway (PPP). Therefore, CO- dependent regulation of PFKFB3 methylation regulates glucose consumption to confirm resistance against oxidative stress for cancer cell survival [111]. HO-1 activities are intensely connected to the modulation of inflammatory and immune responses [116]. HO-1-specific regulatory CD8+ T cells are capable of overwhelming the immune response against cancer cells [117].

MicroRNAs (miRNAs) are small non-coding RNAs that negatively regulate gene expression at the post-transcriptional level. They are involved in cellular development, differentiation, proliferation, and apoptosis and play a significant role in cancer [118]. HO-1 upregulation might be implicated in the directive of microRNA biogenesis by reducing the accessibility of heme, which is the cofactor of DiGeorge critical region 8 (DGCR8), a microRNA processing enzyme [119,120].

Therefore, because modulation of HO-1 in various cancer cells exerts different consequences, determining its various molecular aspects is of particular importance. Overexpression of HO-1 promotes survival and inhibits apoptosis in a renal cancer cell line [121]. Some autophagy-relating molecules, such as beclin-1 or light chain-3B (LC3B), were downregulated by HO-1 overexpression [122]. Based on some studies, different cellular localization of HO-1 can be effective in the final function of this enzyme. HO-1 in the nucleus, through interaction with other proteins, triggers upregulation of AP-1, AP-2, STAT1-3, and Nrf2 and downregulation of NFκB or SP1. In contrast, HO-1 translocation to the nucleus is associated with the loss of HO activity as an enzyme [123]. Hypoxia also can induce the interaction between Nrf2 and HO-1 in the nucleus to contribute to the expression of target antioxidant genes [124]. Based on the information listed, the Nrf2/HO-1axis displays a dual role in cancer, and its up/down-regulation can be used as a novel mechanism in the design of chemotherapy agents.

In the next section, we focus on natural compounds with a modulatory effect on the Nrf2/HO-1 axis and their application in inhibiting the growth and proliferation of lung cancer cells.

## 5. Nrf2/HO-1 Axis Modulating by Natural Antioxidants in Lung Cancer

Lung cancer is the leading cause of cancer deaths worldwide. In 2018, GLOBOCAN estimated 2.09 million new cases (11.6% of total cancer cases) and 1.76 million deaths (18.4% of total cancer deaths); therefore, it is the most frequent cancer and cause of cancer death in men and women combined [125]. Two main forms of lung cancer are NSCLC, non-small-cell lung cancer (about 85% of all lung cancers), and SCLC, small-cell lung cancer (about 15%). Despite development in early detection and standard treatment, NSCLC is often diagnosed at an advanced stage and has a poor prognosis [126,127]. According to huge results, the Nrf2/HO-1 axis exerts an essential role in lung cancer cell growth and proliferation. The Nrf2/HO-1 axis is one of the most pivotal pathways known in the induction of cancer cell chemo-resistance. Inhibition of HO-1 activity caused a significant boost in ROS production in cisplatin-treated A549 cells [128]. Pharmacologic inhibitors of MAPK inhibited the stimulation of HO-1 and Nrf2 expression by cisplatin. Therefore, HO-1 may modify the chemo-sensitivity of A549 cells to cisplatin through the MAPK–Nrf2 pathway [128]. High HO-1 expression in NSCLC is connected with tumor invasiveness and reduced clinical results in NSCLC patients [129,130]. The upregulation of HO-1 in NSCLC displays the main role in chemo-resistance [128,131]. In contrast, overexpression of HO-1 in A549 cancer cells reduced their growth and proliferation [132]. Nrf2 silencing can exert similar consequences in A549 cells [133].

Overexpression of HO-1 in NCI-H292 cells inhibits their proliferation, migration, and angiogenic potential [114]. Lipocalin 2 (LCN2), a multifunctional secretory protein identified as neutrophil gelatinase-associated lipocalin (NGAL), is expressed in various cancers, and depletion of NGAL expression reduced cell growth and proliferation and promoted cell apoptosis [134,135]. NGAL diminution was enough to cause apoptosis of lung adenocarcinoma cells by generating ROS through the inhibition of the Nrf2/HO-1 axis in the presence of *N*-acetylcysteine [136]. Thymidine phosphorylase (TP), a proangiogenic enzyme, is a promising target for anticancer therapy. Overexpression of Nrf2 or HO-1 resulted in the upregulation of TP in NCI-H292 cells. TP-overexpressing NCI-H292 lung tumors in vivo revealed better oxygenation and higher expression of IL-8, IL-1β, and IL-6 [137]. Considering the role of the Nrf2/HO-1 axis in the inhibition or progression of cancer, further in vitro and in vivo studies are needed to elucidate more accurate molecular mechanisms of this pathway.

### In Vitro and In Vivo Targeting of Nrf2/HO-1 Axis by Natural Agents in Lung Cancer

**Figure 6:**
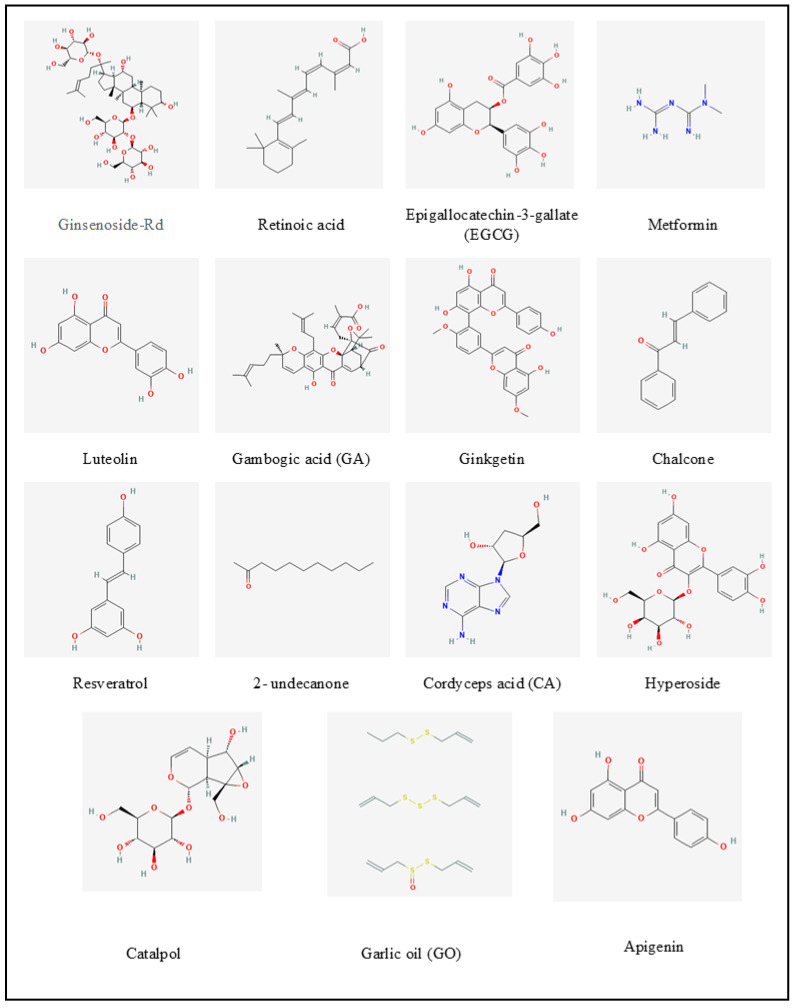
Collection of natural antioxidant compounds with a special effect on the Nrf2/HO-1 axis.

A variety of natural antioxidant compounds have a special effect on the Nrf2/HO-1 axis and lead to the suppression of lung cancer cell growth and proliferation (Figure 6). HO-1 is upregulated in malignant epithelial cells in NSCLC, and its expression is linked with advanced stages of some diseases. As summarized in Table 1, several studies have been conducted to identify the effects of various natural compounds on the Nrf2/HO-1 pathway. In continuing, we review some natural compounds acting on lung cancer through the Nrf2/OH-1 axis.

- **Ginsenoside Rd**, a main active constituent in Panax ginseng, suppresses the growth and propagation of A549 and A549/DDP cells. This component converses cisplatin resistance in A549/DDP cells by reducing the transactivation of Nrf2-related genes [138].
- **Retinoic acid**, a metabolite of vitamin A and modulator of T cell immunity, has a favorable result in the treatment of lung cancer by suppressing Nrf2-related antioxidants in combination with cisplatin. This combination therapy promoted autophagy in cancer cells and had a useful result in clinical trials [139].
- **Epigallocatechin-3-gallate (EGCG)**, the main polyphenol in green tea, is extensively studied as a cancer chemo-preventive agent with prospective anti-cancer effects. As mentioned, HO-1 overexpression has been shown in numerous tumors, prompting survival benefits, aggressiveness, and weak results [129,150,151,152]. The overexpression of HO-1 is closely related to the drug resistance of cancer cells. Activation of Nrf2/HO-1 is considered to mediate cellular resistance to EGCG [153].
- **Metformin** is the most commonly prescribed drug for type 2 diabetes mellitus [154]. It has sensitized NSCLC cells to the EGCG treatment by suppressing the Nrf2/HO-1 axis. A549 xenograft nude mice treatment with EGCG (50 mg/kg, i.p.) and metformin (200μg/mL, dissolved in drinking water) showed notable inhibition of tumor growth rate compared with the untreated counterparts. Besides that, the tumor volume in the nude mice subjected to the combined treatment (metformin plus EGCG) was 9.19 ± 3.14% of the control group [153]. As mentioned, activation of Nrf2/Keap1 signaling in cancer cells results in chemoresistance, inactivating drug-mediated oxidative stress and protecting cancer cells from drug-induced cell death. According to the obtained results, metformin has an inhibitory effect on Nrf2 and inhibits chemo-resistance in cervical and endometrial cancer [155].
- **Luteolin**, a flavonoid extensively distributed in the plant kingdom, has two benzene rings and hydroxyl groups, and this structural specificity contributes to its various biological activities [156]. It suppresses Nrf2 activity by increasing Nrf2 mRNA turnover and sensitizes NSCLC A549 cells to therapeutic drugs [140]. Its use, either alone or in combination with cisplatin, is observed to significantly reduce the growth of xenograft tumors from the A549 cells in athymic nude mice [157].
- **Gambogic acid (GA)** is a natural compound obtained from gamboge, a dry resin secreted from the *Garcinia hanburyi* tree in Southeast Asia. GA inhibits the growth and proliferation of numerous types of human cancer cells, including lung cancer, in vitro and in vivo [158]. This compound, through increasing intracellular ROS in A549 and NCI-H460 cells, can induce apoptosis. Cisplatin (CDDP) and GA combination therapy could suppress NF-*κ*B and mitogen-activated protein kinase (MAPK)/heme oxygenase-1 (HO-1) signaling pathways, which have been confirmed to decrease ROS production and converse CDDP resistance [141].
- **Ginkgetin**, a bioflavonoid obtained from *Ginkgo biloba* leaves, revealed anticancer effects on NSCLC by promoting autophagy. Ferroptosis can be activated by autophagy, which controls redox homeostasis [159]. This compound, through increasing intracellular ROS levels and suppression of the Nrf2/HO-1 axis, can disturb redox hemostasis in DDP-treated cells. Ginkgetin with CDDP also has a synergic cytotoxic effect on NSCLC cells [142].
- **Chalcone** is a common simple scaffold found in many naturally occurring compounds. These natural products and synthetic compounds have shown various remarkable biological activities with clinical potential against various diseases [160]. A series of novel substituted phenyl- (3-methyl-1H-indol-2-yl)-prop-2-en-1-one (indolyl-chalcone) derivatives synthesis and their effects on modulating Nrf2 were investigated. Based on obtained results, one of the synthetic derivatives (3d) displays an effective anti-growth activity by inducing A549 lung cancer cell apoptosis and activating the Nrf-2/HO-1 pathway. In in vivo studies, an A549 xenograft tumor in the chick embryo chorioallantoic membrane (CAM) model proved that indolyl-chalcone repressed tumor growth efficiently by inducing cell apoptosis [143].
- **Resveratrol** (3,5,4′-trihydroxy-trans-stilbene) belongs to the polyphenols’ stilbenoids group, possessing two phenol rings linked to each other by an ethylene bridge [161]. It displays potential anti-carcinogenic activities through HO-1 modulation. The lung adenocarcinoma cell line A549 cells treated with resveratrol (50μM) for 24 h displayed a reduction in the migratory (38% inhibition) and invasive abilities (30% inhibition). It also significantly suppressed HO-1-mediated matrix metalloproteinases (MMP) MMP-9 and MMP-2 expression in lung cancer cells [144]. MMPs are fundamental enzymes in cancer development and are involved in cancer cell metastasis.
- *H. cordata* is a widely used herbal medicine and is also popularly consumed as a healthy vegetable. This medicinal plant and its bioactive compound **2-undecanone** pointedly inhibited B[a]P-induced lung tumorigenesis without triggering apparent systemic toxicity in vivo. This bioactive compound significantly triggered the Nrf2-related antioxidant enzymes, including HO-1 and NQO-1 [145]. *Houttuynia cordata Thunb*. (*H. cordata*) is a prominent medicinal herb in traditional Chinese medicine. Based on some studies, *H. cordata* exerts a variety of pharmacological functions, including antiviral, antitumor, anti-inflammatory, antioxidant, and anti-mutagenic functions [162]. Obtained results display that *H. cordata* and 2-undecanone defend BEAS-2B cells and A/J mice from benzo(a)pyrene (B[a]P)-induced DNA damage.
- **Cordyceps acid (CA)**, a component of *Cordyceps sinensis* extract, exerts numerous pharmacological effects, including antibacterial, antioxidant, and anti-cancer properties [163]. Treatment of A549 tumor-bearing mice with CA (20 and 40 mg/kg) meaningfully reduced the tumor volume, decreased TNF-α, IL-6, and IL-1β, p-NF-κBp65, and increased Nrf2 and HO-1 in the comparison control group [146].
- **Hyperoside** (quercetin-3-O-galactoside) is a flavonol glycoside mainly present in plants of the genera *Hypericum* and *Crataegus* and has several potent pharmacological activities, including anti-inflammatory, antithrombotic, antidiabetic, hepato-protective, and antioxidant effects in various experimental models [164]. AMPK is a very preserved serine/threonine protein kinase comprising a catalytic subunit (α) and two regulatory subunits (β and γ). This kinase exerts a pivotal role in heme oxygenase-1(HO-1) induction. Based on Chen et al., hyperoside prompts A549 cell death via up-regulation HO-1 expression dependent on AMPK activation [147].
- **Catalpol,** an iridoid glucoside contained richly in the roots of the small flowering plant species *Rehmannia glutinosa* Libosch, has been revealed to have antioxidant, anti-inflammation, and anti-apoptosis properties [165]. Based on some results, treatment of lung cancer cells with catalpol remarkably reduce the protein levels of Nrf2 and HO-1 compared with the control group [166].
- **Garlic oil (GO)** is a natural product used for medicinal purposes due to its constituent compounds with several biological effects [167]. It significantly suppressed the 4-(Methylnitrosamino)-1-(3-pyridyl)-1-butanone (NNK)-induced lung cancer in vivo and protected MRC-5 cells from NNK-induced cell damage. It could prompt the expressions of various phase II detoxification enzymes, including NQO-1, glutathione S-transferase alpha 1 (GSTA1), and the antioxidative enzyme HO-1 [148].
- **Apigenin** (4,5,7-trihydroxyflavone; **APG**), as a natural dietary flavonoid, is an effective small molecule inhibitor against Nrf2 and has displayed anticancer activity in various cancers [149]. Due to the limitation of its medicinal use, it is necessary to use suitable carriers to increase the solubility and absorption of this substance. Therefore, nanostructured lipid carriers (NLCs) were used to increase APG efficacy as an Nrf2 inhibitor in combination with docetaxel (DTX) in A549 NSCLC. APG-NLCs had more cytotoxicity and synergistic effect combined with DTX. Treatment of A549 cells with APG-NLCs meaningfully caused a reduction in Nrf2, MRP2, HO-1, and Bcl-2, along with an increase in Bid mRNA levels compared to the other groups [168].

**Figure 7:**
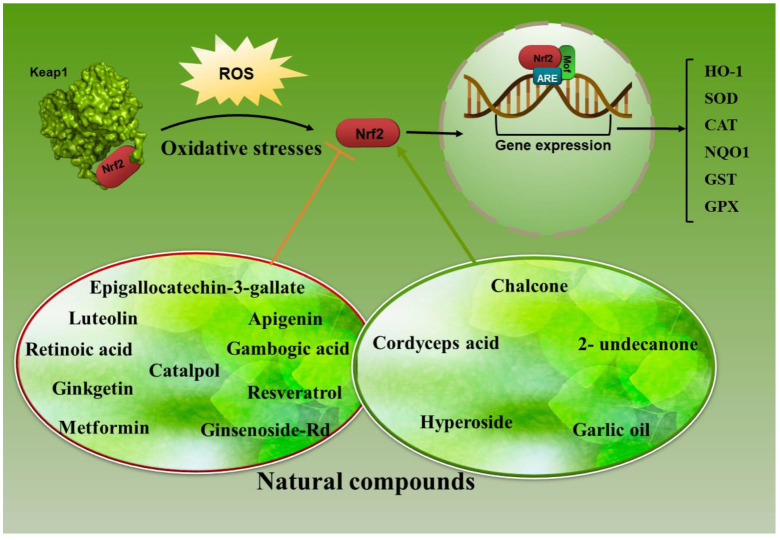
Activation/suppression of the Nrf2-Keap1 pathway and its related genes by various natural compounds has an important role in cancer cell growth and progression.

According to the description provided, natural compounds can modulate oxidative stresses through the Nrf2-Keap1 antioxidative pathway. Due to the dual role of this pathway, cellular conditions and cell type determine the type of cellular response to these natural compounds. Activation/suppression of Nrf2 and its related genes by various natural compounds have an important role in cancer cell growth and progression (Figure 7), and further studies in this field will evolve our understanding of this dual pathway.

## 6. Conclusions and Feature Perspectives

The evolutionary path of the cells creates conditions that they can achieve special adaptations in different situations and continue their growth and proliferation. The Nrf2 pathway, in addition to maintaining cellular oxidative hemostasis, paves the field for various cell processes to ultimately benefit both normal and cancer cells. This pathway, as a master antioxidant pathway, has a complex correlation with various transcription factors as well as some signaling pathways and can exert a dual effect in normal and cancer cells. Some intermediaries are responsible for various features of this pathway. Identifying each loop of this chain can help to understand its performance exactly. Different proteins are regulated by Nrf2, responsible for the dual functions of this pathway. HO-1 is one of the key intermediates in the effects of varied performances of the Nrf2 pathway, which target its effect and can represent a new horizon in dealing with the growth and proliferation of cancer cells and become the basis for designing effective medicines in the treatment of lung cancer.

## Figures and Tables

**Table 1:** Nrf2/HO-1 axis targeting by natural compounds in vitro and in vivo.

Natural Compound	Candidate Cell Model(S)	Mechanism of Action	Ref.
Ginsenoside Rd	A549, A549/DDP.	- Inhibition of cell growth and proliferation - Reducing the expression of Nrf2 and Nrf2-related proteins	[138]
Retinoic acid	A549.	- Suppression of Nrf2 and Nrf2-related genes - Induction of autophagy	[139]
Luteolin	A549, A549 xenograft nude mice.	- Suppresses Nrf2 activity by increasing Nrf2 mRNA turnover - Sensitizing cancer cells to therapeutic drugs - Reduce the growth of xenograft tumors	[140]
Gambogic acid	A549, NCI-H460.	- Inhibition of cell growth and proliferation - Increasing intracellular ROS and induction of apoptosis - Inhibition of NF-*κ*B and MAPK/HO-1 signaling in combination with cisplatin	[141]
Ginkgetin	A549, NCI-H460, SPC-A-1, A549 xenograft nude mice.	- Suppression of the Nrf2/HO-1 axis - Increasing intracellular ROS - Induction of autophagy - Synergic cytotoxic effect on cells in combination with cisplatin	[142]
Chalcone	A549, A549 xenograft tumor in CAM model.	- Activation of the Nrf-2/HO-1 axis - Increases oxidative stress and induction of apoptosis - Inhibits the tumor xenograft growth	[143]
Resveratrol	A549.	- Reduction in the migratory and invasive abilities of cells - Suppressed HO-1-mediated MMP-9 and MMP-2 expression	[144]
2-Undecanone	B[a]P-induced lung cancer mouse model.	- Inhibition of B[a]P-induced lung tumorigenesis in a mouse model - Increased protein levels of Nrf2, HO-1 and NQO-1 - Decreased mean tumor numbers in the mouse model	[145]
Cordyceps acid	A549 tumor-bearing mice.	- Reduction in the tumor volume - Decreased TNF-α, IL-6, and IL-1β, p-NF-κBp65 - Increased Nrf2 and HO-1 activity	[146]
Hyperoside	A549.	- Promotion of cell death via up-regulation of HO-1 expression	[147]
Garlic oil	NNK-induced lung cancer mouse model.	- Suppression of NNK-induced lung cancer - Protection of MRC-5 normal cells from NNK-induced cell damage - Induction of NQO-1, GSTA1 and HO-1 expression	[148]
Apigenin	A549.	- Decrease in Nrf2, MRP2, HO-1, and Bcl-2 mRNA level - Increase in Bid mRNA - More cytotoxicity and synergistic effect combined with DTX	[149]

## Data Availability

The data is contained within the article.
